# 基于高效液相色谱定量指纹图谱和液相色谱-质谱联用定量的酸枣仁提取物质量考察

**DOI:** 10.3724/SP.J.1123.2021.06019

**Published:** 2021-09-08

**Authors:** Xiujie GUO, Haoqiu LI, Haotian FENG, Huawen QI, Lu ZHANG, Wei XU, Yanjuan WU, Chaoran WANG, Xinmiao LIANG

**Affiliations:** 1.中国科学院大连化学物理研究所, 辽宁 大连 116023; 1. Dalian Institute of Chemical Physics, Chinese Academy of Sciences, Dalian 116023, China; 2.中科院大化所中国医药城生物医药创新研究院, 江苏 泰州 225300; 2. DICP-CMC Innovation Institute of Medicine, Taizhou 225300, China; 3.内蒙古乳业技术研究院有限责任公司, 内蒙古 呼和浩特 010110; 3. Inner Mongolia Dairy Technology Research Institute Co. Ltd., Huhehaote 010110, China; 4.内蒙古伊利实业集团股份有限公司, 内蒙古 呼和浩特 010110; 4. Inner Mongolia Yili Industrial Group Co. Ltd., Huhehaote 010110, China

**Keywords:** 高效液相色谱, 质谱, 定量指纹图谱, 酸枣仁提取物, 雷达图, high performance liquid chromatography (HPLC), mass spectrometry (MS), quantitative fingerprint, *Ziziphi Spinosae Semen* extracts, radar-gram

## Abstract

酸枣仁具有显著的改善睡眠和抗焦虑等作用,其提取物在助眠类功能食品开发中应用前景广阔。但目前市场上酸枣仁提取物质量参差不齐,缺乏统一标准,企业在使用时面临较大的质量风险,因此亟须建立一种准确、全面的内控质量评价方法。针对酸枣仁提取物中黄酮和皂苷两类主要活性成分紫外响应差异巨大且水提物中皂苷成分含量低的问题,该研究建立了酸枣仁水提物HPLC定量指纹图谱方法,共标定了8个共有峰。通过对照品指认、文献比对以及高效液相色谱-四极杆飞行时间质谱数据解析,8个共有峰均为黄酮类化合物,该方法可同时实现7种黄酮成分的半定量对比分析和斯皮诺素的含量测定;采用超高效液相色谱-三重四极杆质谱,在正离子模式下,以多反应监测扫描方式可实现酸枣仁皂苷A和B的含量测定;最终以雷达图展现上述10种成分的半定量和定量数据。应用上述方法,该研究对比分析了实验室自制的3批酸枣仁水提物和15家供应商的15批提取物样品。结果显示,实验室自制的3批酸枣仁水提物虽然原料来自不同饮片企业,但总体差异性不大,而不同厂家提供的酸枣仁提取物样品成分含量差异巨大,提示不同厂家存在辅料稀释、理枣仁掺假和醇提或纯化富集等情况。该法为企业制定内控质量标准和筛选合格供应商提供了依据。

## Abstract (en)

*Ziziphi Spinosae Semen* is the dried seeds of *Ziziphus jujuba* Mill. var. *spinosa* (Bunge) Hu ex H. F. Chou, and its extract has broad application prospects in the development of sleep-aid functional foods. However, the quality parameters of *Ziziphi Spinosae Semen* extracts currently available in the market are not uniform and there is a lack of unified standards. Therefore, it is important to establish an accurate and comprehensive method for quality evaluation. In view of the problems that the UV responses of flavonoids and saponins in the *Ziziphi Spinosae Semen* extracts vary dramatically and the saponin content in *Ziziphi Spinosae Semen* water extract is very low, high performance liquid chromatography (HPLC) was used to establish the fingerprint and quantify spinosin. The separation was carried out on a Waters XSelect HSS C18 column (250 mm×4.6 mm, 5 μm), and the mobile phase was acetonitrile-0.1% (v/v) phosphoric acid aqueous solution for gradient elution. The eight common peaks in the fingerprint of the *Ziziphi Spinosae Semen* extracts, identified by HPLC-quadrupole time-of-flight mass spectrometry, were attributed to flavonoids by reference substance identification, literature comparison, and high-resolution mass spectrometry data analysis. Semi-quantitative analysis of seven flavonoids and quantitative analysis of spinosin were conducted using the established HPLC quantitative fingerprint. The contents of jujuboside A and jujuboside B were determined by ultra-high performance liquid chromatography-triple quadrupole mass spectrometry. Chromatographic separation was performed on a Waters ACQUITY UPLC BEH C18 column (50 mm×2.1 mm, 1.7 μm) by gradient elution using a mobile phase of acetonitrile-0.1%(v/v) formic acid aqueous solution. The target compounds were analyzed in multiple reaction monitoring mode with positive electrospray ionization.

The semi-quantitative and quantitative data of the above-mentioned 10 components are displayed in the form of radar. Using the above methods, three batches of *Ziziphi Spinosae Semen* water extracts prepared in the laboratory and 15 batches of extract samples obtained from 15 suppliers were analyzed and compared. The results showed that although the raw materials of three batches *of Ziziphi Spinosae Semen* water extracts prepared in the laboratory were from different enterprises, the overall difference was not significant. However, the component contents of the samples provided by different manufacturers were greatly different, suggesting that there are some problems associated with the different manufacturers, such as dilution of excipients, adulteration of *Ziziphi Mauritianae Semen*, alcohol extraction, purification, and enrichment. For example, the representative composition contents in the *Ziziphi Spinosae Semen* extracts obtained from manufacturers B, C, E, F, G, H, I, and O were low, which were approximately 1/10 of corresponding contents in the normal water extracts prepared in the laboratory. It is speculated that to reduce the unit price of the product, the manufacturer used fewer raw materials or a large number of auxiliary materials to dilute the *Ziziphi Spinosae Semen* extracts. The contents of some flavonoids in the *Ziziphi Spinosae Semen* extract from manufacturer N were slightly higher than that in the self-preparation *Ziziphi Spinosae Semen* water extract, but it did not contain jujuboside A; thus, it was speculated that the *Ziziphi Mauritianae Semen* might be used for extraction. The contents of 10 components in the *Ziziphi Spinosae Semen* extract obtained from manufacturer D were all higher than the corresponding ones in the self-preparation *Ziziphi Spinosae Semen* water extract. Combined with the quality label of total saponin content > 20% and poor water solubility, it was speculated that the product might be prepared by alcohol extraction or purified and enriched by using resin. These results provided the basis for the enterprise to establish internal control quality standards for *Ziziphi Spinosae Semen* extracts and to select qualified suppliers.

酸枣仁为鼠李科植物酸枣*Ziziphus jujuba* Mill. var. *spinosa* (Bunge) Hu ex H. F. Chou的干燥成熟种子,具有养心补肝,宁心安神等功效^[1,2]^,是我国最著名的安神类中药材之一,也是卫生部颁布的第一批药食同源中药材,被广泛应用于“枣仁安神颗粒”、“百乐眠胶囊”和“太太静心口服液”等中成药或保健品^[3,4]^。酸枣仁的主要活性成分为以斯皮诺素为代表的黄酮类和以酸枣仁皂苷A和B为代表的皂苷类等。研究表明,这些活性成分不仅具有镇静催眠作用,还具有神经元保护作用,可抗焦虑、抗抑郁、改善记忆和治疗老年痴呆等^[5,6,7]^。随着现代社会工作节奏的加快,生活压力的增加,患有睡眠障碍问题的人群越来越多,酸枣仁水提物作为一种有效的助眠原料也正被日益广泛地应用到各类助眠产品的开发中,获得了极大的关注。然而,当前市场上酸枣仁提取物缺少统一标准,市场产品良莠不齐,存在理枣仁掺假、辅料稀释增重或采用醇提等不合规生产工艺提高成分含量等一系列问题,导致企业在应用酸枣仁提取物开发功能性食品时,难以评估供应商原料的真假优劣,面临较大的质量风险。为此,企业亟须建立一种准确、全面的分析方法对酸枣仁提取物进行质量评价。

2020版《中国药典》对酸枣仁药材分别采用两个液相色谱方法对斯皮诺素和酸枣仁皂苷A进行含量测定,其中酸枣仁皂苷A的含量测定使用蒸发光散射检测器(ELSD)检测。斯皮诺素在掺假品理枣仁中含量更高,而酸枣仁皂苷A在理枣仁中几乎没有,酸枣仁皂苷B在酸枣仁中的含量也远高于理枣仁,因此两个皂苷成分可以作为两者区分的特征成分^[8,9]^。目前,针对酸枣仁提取物含量测定的文献报道较少,且多针对醇提物中酸枣仁皂苷A和B的测定^[10]^,或者是采用树脂富集后进行总皂苷测定^[11]^。但由于酸枣仁皂苷本身含量较低(饮片要求含量不低于0.03%),水提过程中皂苷转移率低,且在生产过程中往往添加辅料辅助干燥或进行稀释,针对药材或醇提工艺开发的HPLC-ELSD含量测定方法灵敏度不足以适用于提取物的检测。为此,本文建立了超高效液相色谱-三重四极杆质谱(UPLC-QqQ-MS/MS)法来测定酸枣仁水提物中皂苷A、B含量,同时采用定量指纹图谱的方法,建立了斯皮诺素的含量测定及其他7种成分的峰面积半定量对比方法,最终将这10种成分的含量差异统一用雷达图形象展示,对不同厂家的酸枣仁提取物进行比较研究,为酸枣仁提取物的内控质量标准建设和供应商筛选提供更多依据。

## 1 实验部分

### 1.1 仪器、试剂与材料

Waters Alliance高效液相色谱系统,包括2695梯度泵、2998二极管阵列检测器、自动进样器、柱恒温系统和Empower色谱工作站;岛津超高效液相色谱(UHPLC)系统,配SPD-20A紫外检测器,与AB SCIEX X500系列四极杆飞行时间(QTOF)质谱仪联用;Waters I-Class Xevo TQ-XS超高效液相色谱-三重四极杆质谱仪;MS204TS电子分析天平(梅特勒-托利多有限公司)。

斯皮诺素对照品(批号DST191025-060,规格20 mg,含量92.7%)、酸枣仁皂苷A对照品(批号DST200510-058,规格20 mg,含量91.7%)和酸枣仁皂苷B对照品(批号DST210412-059,规格20 mg,含量87.2%)购自成都德思特生物技术有限公司。3批酸枣仁饮片分别购自四川新荷花中药饮片股份有限公司、北京宏济药业有限公司和盛实百草药业有限公司,并经中国科学院大连化学物理研究所杨小平高级工程师鉴定为鼠李科植物酸枣*Ziziphus jujuba* Mill. var. *spinosa* (Bunge) Hu ex H. F. Chou的干燥成熟种子。

酸枣仁提取物为自制或购自不同厂家,具体信息见表1。乙腈(色谱级)、甲醇(色谱级)均购自Sigma-Aldrich。磷酸(色谱级)、甲酸(色谱级)购自Aladdin。实验室用水来自美国Milli-Q超纯水净化系统。

**表1:** 酸枣仁提取物样品信息

No.	Manufacturer	Batch	Standard
1	self-preparation	STW2006093	-
2	self-preparation	STW078190801	-
3	self-preparation	STW19072202	-
4	A	HS-A1102	jujuboside A+jujubo-
			side B≥ 0.19%
5	B	ZLSC2020071505	10:1 (raw materials:
			extracts)
6	C	T2004223	5:1 (raw materials:
			extracts)
7	D	CO360191002	total saponins≥ 20%
8	E	20200301ZF	total saponins≥ 0.1%
9	F	ZJ100-200506	-
10	G	NSMP20080381-	1:1 (raw materials:
		ZZP-10	extracts)
11	H	19052204	-
12	I	T20041701	jujuboside A≥ 0.03%
13	J	20051901	total saponins≥ 2%
14	K	S1042009034	-
15	L	201001	-
16	M	20052601	jujuboside A+juboside
			B≥0.19%
17	N	200711	-
18	O	201203	-

-: no quality standard

### 1.2 样品制备

1.2.1 酸枣仁水提物

称取40 g酸枣仁饮片至圆底烧瓶,加水400 mL,浸泡30 min,回流提取1 h,滤过,滤液转移至1000 mL容量瓶中,滤渣加400 mL水二次回流提取1 h,滤过,滤液转移至前述容量瓶中,冷却至室温后定容,摇匀,取500 mL酸枣仁水提液,浓缩至100 mL,冷冻干燥,得到自制酸枣仁水提物。

1.2.2 定量指纹图谱供试品溶液

取酸枣仁提取物,精密称取约1 g置于25 mL容量瓶中,加50%(v/v)甲醇水溶液约15 mL,振荡,超声30 min使全部溶解或溶散,冷却至室温后用50%(v/v)甲醇水溶液定容,过0.22 μm滤膜,即得。

1.2.3 酸枣仁皂苷含量测定供试品溶液

取酸枣仁提取物,精密称取约0.1 g置于100 mL容量瓶中,加50%(v/v)乙腈水溶液约50 mL,振荡,超声20 min使全部溶解或溶散,冷却至室温后用50%(v/v)乙腈水溶液定容,过0.22 μm滤膜,即得。

### 1.3 对照品储备液的制备

取斯皮诺素、酸枣仁皂苷A、酸枣仁皂苷B对照品各10 mg,分别置于10 mL容量瓶中,加甲醇溶解并稀释至刻度,摇匀,分别作为斯皮诺素、酸枣仁皂苷A、酸枣仁皂苷B储备液。

### 1.4 分析条件

1.4.1 HPLC—用于建立对照指纹图谱和特征峰半定量及斯皮诺素含量测定

Waters Alliance HPLC系统,Waters XSelect HSS C18(250 mm×4.6mm, 5 μm)色谱柱,流动相乙腈(A)-0.1%磷酸水溶液(B)洗脱,0~5 min, 10%A~15%A; 5~25 min, 15%A~20%A; 25~35 min, 20%A~30%A; 35~40 min, 30%A~95%A; 40~46 min, 95%A;流速为1.0 mL/min;进样量为10 μL;柱温为40 ℃;检测波长为337 nm。

1.4.2 HPLC-QTOF-MS—用于对照指纹图谱中特征峰的鉴定

岛津UHPLC系统,与AB SCIEX X500系列QTOF质谱仪联用,LC条件中流动相为乙腈(A)-0.1%甲酸水溶液(B),其余条件同1.4.1节。离子源:ESI;负离子模式;气帘气:241 kPa; Gas 1: 379 kPa; Gas 2: 379 kPa;温度:550 ℃;离子化压力:-4500 V,去簇电压:-80 V;全扫描范围:*m/z* 50~1500;裂解电压:-40 V。CE Spread: 20 V。

1.4.3 UPLC-QqQ-MS—用于皂苷A、B的定量测定

Waters I-Class Xevo TQ-XS UPLC-QqQ-MS仪,Waters ACQUITY UPLC BEH C18(50 mm×2.1 mm, 1.7 μm)色谱柱。流动相为乙腈(A)-0.1%甲酸水溶液(B);梯度洗脱:0~2 min, 19%A~23%A; 2~4 min, 23%A~95%A; 4~7 min, 95%A; 7~7.1 min, 95%A~19%A; 7.1~10 min, 95%A~19%A,流速为0.3 mL/min,柱温为30 ℃;进样量为2 μL。离子源:ESI;正离子模式;碰撞气,氩气;雾化气,氮气;加热气,氮气;毛细管电压3.00 kV;源温度150 ℃, MRM模式监测,皂苷质谱参数见表2。

**表2:** 酸枣仁皂苷A和B的质谱参数

Compound	Retention time/min	[M+H]^+^ (m/z)	Daughter ion (m/z)	CV/ V	CE/ eV
Jujuboside A	3.26	1207.64	455.41^*^	60	62
			85.10		22
Jujuboside B	3.38	1045.58	455.41^*^	60	64
			85.10		26

* Quantitative ion. CV: capillary voltage; CE: collision energy.

## 2 结果与讨论

### 2.1 指纹图谱的建立

取3批自制酸枣仁水提物,按1.2.2节的方法制备HPLC指纹图谱供试品溶液,按1.4.1节的方法依次进样检测,将3批自制酸枣仁水提物样品的色谱图全部导入《中药色谱特征图谱相似度评价系统软件》(2012版),以第一批自制酸枣仁水提物(STW2006093)的图谱为校正参照,使用中位数进行自动匹配,加以多点校正,可识别8个主要共有色谱峰,得到酸枣仁水提物的对照谱图(见图1)。选择出峰时间稳定且相对居中的斯皮诺素峰(3号峰)作为参比峰。

**图1:**
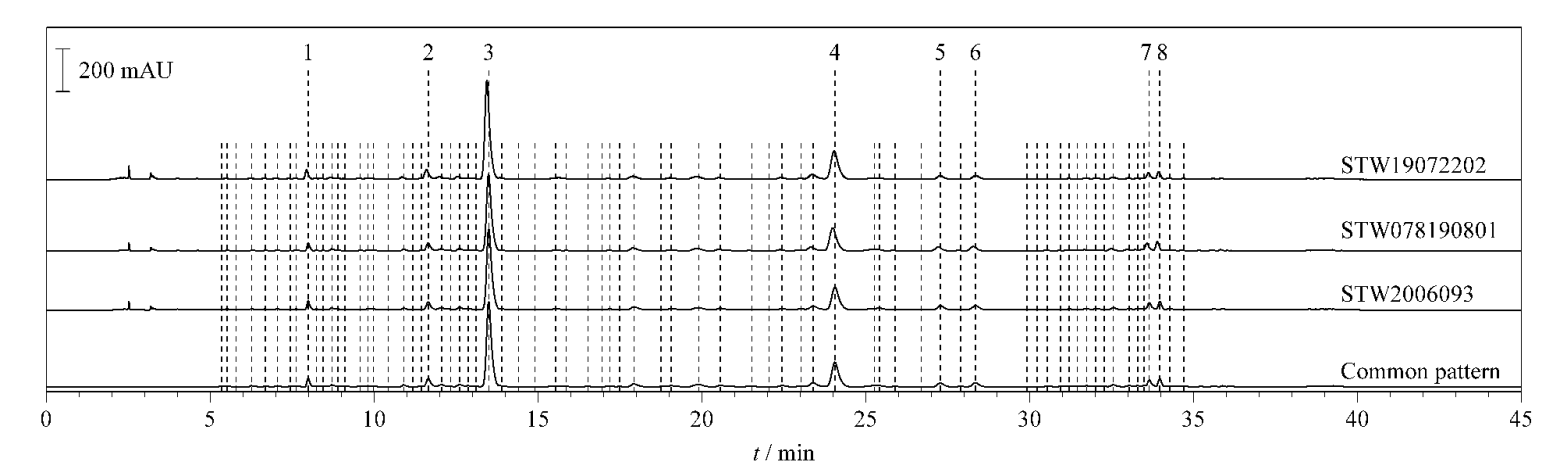
酸枣仁水提物HPLC指纹图谱及其共有模式

取批号为STW2006093的自制酸枣仁水提物,制备供试品溶液,以HPLC-QTOF-MS分析,采集质谱数据,通过比对精确相对分子质量和特征碎片离子,结合软件推断的分子式及文献报道^[12,13]^,鉴定出酸枣仁水提物8个共有峰的结构,结果见表3。

**表3:** 酸枣仁水提物特征峰的鉴定

Peak No. in Fig. 1	Ionization mode	[M-H]^-^ (m/z)	Fragment ions (m/z)	Compound
1	ESI^-^	593.1229	503.1137, 473.1072, 383.0766, 353.0653	vicenin-2
2	ESI^-^	593.1250	413.0817, 293.0450	glucosylvitexin
3	ESI^-^	607.1390	445.1130, 427.1019, 307.0610	spinosin
4	ESI^-^	783.1782	427.103, 307.0614	6″'-feruloylspinosin
5	ESI^-^	942.2246	762.1987, 427.0920, 293.0452	6″-O-(3-Glc-indole-acetyl) spinosin or its isomer
6	ESI^-^	942.2246	762.2038, 607.1740	6″-O-(3-Glc-indole-acetyl) spinosin or its isomer
7	ESI^-^	1118.2650	293.0479	6″-O-(3-Glc-indole-acetyl)-6″'-feruloylspinosin or its isomer
8	ESI^-^	1118.3100	/	6″-O-(3-Glc-indole-acetyl)-6″'-feruloylspinosin or its isomer

/: no fragment ions were detected.

取15批不同厂家酸枣仁提取物,采集得到HPLC指纹图谱(见图2)并进行相似度分析。15批不同厂家的酸枣仁提取物指纹图谱与共有模式对照指纹图谱相似度分别为0.992(A), 0.947(B), 0.960(C), 0.991(D), 0.976(E), 0.966(F), 0.957(G), 0.978(H), 0.935(I), 0.961(J), 0.989(K), 0.104(L), 0.989(M), 0.950(N), 0.980(O)。相似度结果显示,除厂家L外,14批不同厂家酸枣仁提取物的相似度均在0.9以上,但从色谱图上看,这些提取物样品在成分组成和含量上有着显著差异。

**图2:**
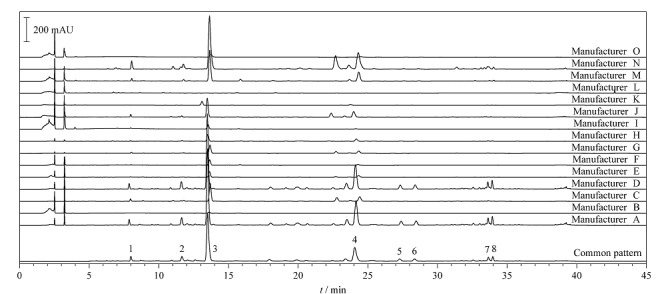
15批酸枣仁提取物的HPLC指纹图谱

### 2.2 指纹图谱方法学考察^[14]^

精密度 取批号为STW2006093的自制酸枣仁水提物,制备供试品溶液,连续进样6次,8个特征峰的相对保留时间RSD均小于1%,相对峰面积RSD小于5%,表明仪器精密度良好。

稳定性 取批号为STW2006093的自制酸枣仁水提物,制备供试品溶液,分别于0、6、12、18、24 h注入液相色谱仪,8个特征峰的相对保留时间RSD均小于1%,相对峰面积RSD小于5%,表明供试品溶液24 h内稳定性良好。

重复性 取批号为STW2006093的自制酸枣仁水提物,制备6份供试品溶液,进样测定,8个特征峰的相对保留时间RSD均小于1%,相对峰面积RSD小于5%,表明该方法重复性良好。

### 2.3 斯皮诺素方法学考察与含量测定

检出限与定量限 精密吸取斯皮诺素储备液适量,逐级稀释后进行测定,以信噪比(*S/N*)为3~10作为方法的检出限(LOD),以*S/N*为10~20作为方法的定量限(LOQ),得出斯皮诺素的检出限为0.6 mg/L,定量限为1.9 mg/L。

线性关系 精密吸取斯皮诺素储备液适量,配制成5个质量浓度系列的对照品溶液,进行测定,以斯皮诺素质量浓度(*X*)为横坐标,峰面积(*Y*)为纵坐标,绘制标准曲线,得线性回归方程、相关系数(*r*^2^)和线性范围,结果见表4。

**表4:** 斯皮诺素、酸枣仁皂苷A和B的线性关系和线性范围

Compound	Regression equation	r^2^	Linear range/(mg/L)	
Spinosin	Y=26592849.20X-91407.22	0.9998	19300.14-	386002.80
Jujuboside A	Y=167.702X+88.2103	0.9995	4.61-	1845.00
Jujuboside B	Y=316.106X+272.036	0.9992	4.48-	1791.09

*Y*: peak area; *X*: mass concentration, mg/L.

精密度 取斯皮诺素对照品溶液,连续进样6次,测得斯皮诺素峰面积的RSD值为0.38%,表明仪器精密度良好。

稳定性 取批号为STW2006093的自制酸枣仁水提物,按照1.2.2节下样品处理方法制备供试液,在制备后的0、6、12、18、24 h分别进行HPLC测定,得到斯皮诺素峰面积的RSD为0.64%,表明供试品溶液在24 h内稳定性良好。

重复性 取同一自制酸枣仁水提物6份(批号STW2006093),分别制备供试液,测定HPLC谱图,记录斯皮诺素的峰面积,计算6份平行样中斯皮诺素含量RSD为0.45%,表明本方法重复性较好。

加样回收率 取自制酸枣仁水提物6份(批号STW2006093),每份0.5 g,精密称定,置于25 mL容量瓶中,加入斯皮诺素1 mg,加50%(v/v)甲醇水溶液约15 mL,振荡,超声30 min使全部溶解或溶散,冷却至室温后用50%(v/v)甲醇水溶液定容,过0.22 μm滤膜后进行HPLC测定,结果显示,斯皮诺素的回收率为90.70%, RSD为1.16%。

样品含量测定 对3批自制酸枣仁水提物和来自15个不同厂家的酸枣仁提取物进行分析,测定斯皮诺素的含量,结果见表5。结果显示,18批样品中斯皮诺素含量在0~0.62%之间,含量测定的RSD均小于2%。

**表5:** 酸枣仁提取物中斯皮诺素、酸枣仁皂苷A和B的含量及RSD (*n*=3)

No.	Manufacturer	Contents (RSDs)/%		
		Spinosin	Jujuboside A	Jujuboside B
1	self-preparation-STW2006093	0.43 (0.20)	0.16 (0.83)	0.08 (1.31)
2	self-preparation-STW078190801	0.40 (0.53)	0.18 (0.29)	0.09 (0.81)
3	self-preparation-STW19072202	0.51 (0.53)	0.18 (0.65)	0.09 (0.10)
4	A	0.24 (0.18)	0.09 (1.37)	0.13 (0.70)
5	B	0.01 (0.47)	ND	0.01 (0.38)
6	C	0.14 (0.11)	ND	ND
7	D	0.62 (0.23)	0.42 (0.47)	0.22 (0.36)
8	E	0.04 (0.85)	0.01 (0.13)	0.01 (0.28)
9	F	0.05 (1.82)	0.01 (0.20)	0.04 (0.73)
10	G	0.06 (0.14)	ND	ND
11	H	0.05 (0.43)	0.02 (1.17)	0.01 (1.88)
12	I	0.03 (0.23)	0.01 (0.51)	0.03 (0.57)
13	J	0.17 (0.36)	0.01 (0.39)	0.02 (0.64)
14	K	0.03 (1.15)	0.01 (0.48)	0.01 (0.64)
15	L	ND	ND	ND
16	M	0.25 (0.57)	0.08 (0.76)	0.12 (0.97)
17	N	0.52 (0.12)	ND	0.01 (0.83)
18	O	0.03 (1.97)	0.01 (0.88)	0.01 (0.24)

ND: not detected.

### 2.4 酸枣仁皂苷A和B方法学考察与含量测定

检出限和定量限 分别精密吸取酸枣仁皂苷A和B储备液适量,逐级稀释进样测定,以*S/N*为3~10作为方法的LOD,以*S/N*为10~20作为方法的LOQ,得出酸枣仁皂苷A的LOD为0.007 μg/L,LOQ为0.02 μg/L,酸枣仁皂苷B的LOD为0.007 μg/L,LOQ为0.02 μg/L。

线性关系 精密吸取酸枣仁皂苷A和B储备液适量,配制成7个质量浓度的系列混合对照品溶液,进样测定,以质量浓度(*X*)为横坐标,峰面积(*Y*)为纵坐标,得线性回归方程、*r*^2^和线性范围,结果见表4。

精密度 取酸枣仁皂苷A和B对照品混合溶液,连续进样6次测定,得到酸枣仁皂苷A和B峰面积的RSD分别为0.70%和3.29%,表明仪器精密度良好。

稳定性 取自制酸枣仁水提物(批号STW2006093),制备供试液,在制备后的0、6、12、18、24 h分别进行测定,得到酸枣仁皂苷A和B峰面积的RSD分别为1.23%和1.89%,表明供试品溶液在24 h内稳定性良好。

重复性 取同一自制酸枣仁水提物6份(批号STW2006093),制备供试液,进样测定,记录酸枣仁皂苷A和B的峰面积,计算6份平行样中酸枣仁皂苷A和B含量的RSD分别为2.35%和1.88%,表明本方法重复性较好。

加样回收率 取自制酸枣仁水提物6份(批号STW2006093),每份0.05 g,精密称定,置于100 mL容量瓶中,加入酸枣仁皂苷A 0.04 mg,酸枣仁皂苷B 0.02 mg,加50%(v/v)乙腈水溶液约50 mL,振荡,超声20 min,冷却至室温后用50%(v/v)乙腈水溶液定容,过0.22 μm滤膜后进行测定,结果显示,酸枣仁皂苷A和B的回收率分别为94.14%和91.41%, RSD分别为1.27%和2.38%。

样品含量测定 通过对3批自制酸枣仁水提物和15批不同厂家酸枣仁提取物进行分析,测定酸枣仁皂苷A和B的含量,含量测定结果见表5。结果显示,18批样品中酸枣仁皂苷A含量在0~0.42%之间,酸枣仁皂苷B含量在0~0.22%之间。

### 2.5 雷达图分析

雷达图分析方法是基于一种形似导航雷达显示屏上的图形而构建的一种多变量对比分析技术。在雷达图中,每组评价指标都有一个独立的单一数值轴,坐标轴呈辐射状分布在中心点周围,把同一评价对象的不同指标数据值在坐标轴上的点用折线连接起来所形成的多边形就是雷达图^[15,16,17]^。为了更加直观地反映不同厂家酸枣仁提取物的质量差异,本研究采用雷达图对酸枣仁提取物质量进行对比评价。使用开源软件plotlyjs网页绘图库(Plotly公司)制作雷达图。采用3批自制的酸枣仁水提物平均值作为基准进行参照(雷达图中红框),选择10个代表性成分进行评价,其中斯皮诺素、酸枣仁皂苷A和酸枣仁皂苷B采用含量测定结果进行比例折算,定量指纹图谱中其他7个黄酮共有峰采用相对峰面积进行比例折算,10个成分的含量对比雷达图见图3。从雷达图中可以清晰地看到,市场提取物不仅与实验室采用合格饮片自制的水提物含量差距较大,不同厂家之间的成分组成和含量差异也非常大,其中厂家B、C、E、F、G、H、I、O的酸枣仁提取物中代表性成分含量较低,约为实验室正常水提含量的1/10,推测厂家为降低产品单价,酸枣仁投料较少或使用了大量辅料进行稀释,而厂家L几乎未检出任何酸枣仁代表性成分,产品真实性存在较大问题。厂家D的10个成分含量都超过自制酸枣仁水提物,结合总皂苷含量>20%的质量标识和较差的水溶性,推测可能是采用醇提法制备并经树脂纯化富集的产品;厂家N的提取物中部分黄酮类成分含量略高于自制酸枣仁水提物,但不含酸枣仁皂苷A,推测可能提取用原料为酸枣仁的伪品理枣仁^[18]^。只有厂家A的各成分含量与自制水提物均较为接近,且酸枣仁皂苷A和B都有足够的含量,提示其使用的原料和工艺都较为合规。上述结果表明,由于缺乏统一的质量标准要求,各厂家的酸枣仁提取物所用原料和生产工艺可能存在巨大的差异,导致其产品差异性十分巨大,酸枣仁提取物应用企业需要建立严格的内控质量评价体系,对这些原料进行筛选,合理应用。

**图3:**
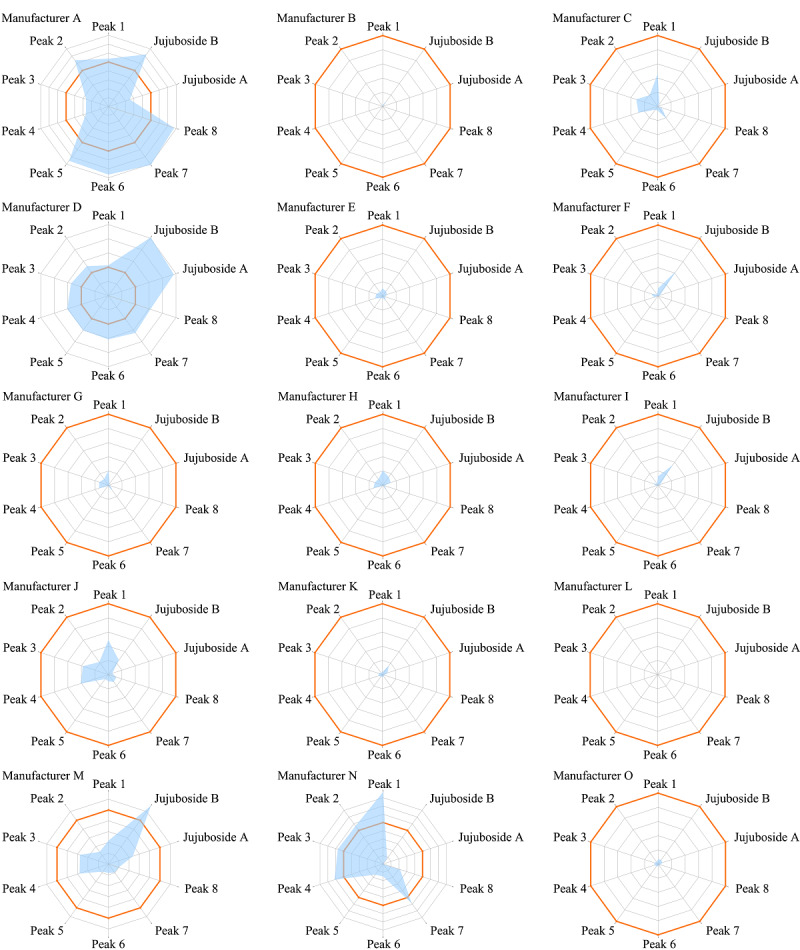
15批酸枣仁提取物雷达图

## 3 结论

本文建立了8个共有黄酮峰为代表的酸枣仁水提物HPLC定量指纹图谱,并通过液相色谱-四极杆飞行时间质谱联用鉴定了共有峰的可能成分。结合斯皮诺素、酸枣仁皂苷A和B定量结果,以及定量指纹图谱中其他7个共有峰的相对定量结果进行雷达图评价。结果显示,不同厂家提供的酸枣仁提取物存在非常大的差异,对比实验室按照正常水提工艺的样品,可能存在大量辅料稀释甚至不含有效成分,以及理枣仁掺假、醇提富集等情况。本文所建立的HPLC定量指纹图谱结合液相色谱-质谱联用定量分析方法,可全面、系统地应用雷达图呈现酸枣仁提取物的质量差异,为酸枣仁提取物应用企业根据自身产品分类和市场定位建立合理内控质量标准并筛选合格供应商提供重要参考。
